# A159 RISK OF TOTAL METACHRONOUS ADVANCED NEOPLASIA IN PATIENTS WITH SERRATED LESIONS AND INFLAMMATORY BOWEL DISEASE

**DOI:** 10.1093/jcag/gwac036.159

**Published:** 2023-03-07

**Authors:** E Medawar, R Djinbachian, I Popescu Crainic, P Lakatos, A Barkun, E -J Bernard, D von Renteln

**Affiliations:** 1 Université de Montréal, Montreal; 2 University of Ottawa, Ottawa; 3 McGill University, Montreal, Canada

## Abstract

**Background:**

The risk of total metachronous advanced neoplasia (TMAN) in patients with serrated lesions (SL) and IBD is unknown. It is also unclear whether colonic inflammation in IBD contributes to serrated neoplasia.

**Purpose:**

Study aim was to compare the risk of TMAN at surveillance colonoscopies in patients with SL and IBD to patients with SL without IBD. We also sought to compare IBD severity in patients with IBD and SL in a colonic area involved with IBD (SL-IA) to patients with IBD and SL in an uninvolved area (SL-UA).

**Method:**

A cohort study was conducted. Through pathology database search, we identified 2428 patients with endoscopically resected SL, defined as sessile serrated lesion (SSL), traditional serrated adenoma (TSA) or IBD and serrated epithelial change (SEC), between 2010 and 2019 at the University of Montreal Hospital Center. We included patients aged 45-75 without polyposis syndromes and excluded patients with a history of CRC, first surveillance <12 months after complete index, sigmoidoscopy at index, or no follow-up. Patient files were reviewed for demographic data, IBD severity, and findings at index and follow-up. Follow-up was continued until TMAN or last colonoscopy within 10 years. Primary outcome was the risk of TMAN (defined as advanced adenoma (AA), advanced serrated lesion (ASL) or CRC) in a surveillance colonoscopy within 10 years from index. Secondary outcomes were the risk of metachronous AA and ASL, and IBD severity in SL-IA and SL-UA. Continuous and categorical variables were compared using the student t, Pearson’s chi-squared, or Mann-Whitney tests. We performed univariate and multivariate Cox regressions, with hazard ratios (HR) and 95% confidence intervals.

**Result(s):**

In the metachronous outcomes analysis, 440 patients with SL (mean age 61.8 y., 51.6% male, 424 SSL, 16 TSA) were eligible, and 37 with SL and IBD were eligible (mean age 60.9 y., 54.1% male, 30 SSL, 6 SEC, 1 TSA). Compared to patients without IBD, IBD patients were less likely to have synchronous adenomas (16.2% vs 41.6%, p<0.05), had less SLs ≥10 mm (24.3% vs 46.8%, p<0.05), and had a similar risk of metachronous TMAN (HR=0.92 [0.44–1.90]), AA (HR=0.53 [0.13–2.12]) and ASL (HR=1.03 [0.44–2.41]). In the comparison of SL-UA and SL-IA, 56 patients with IBD were eligible, with 21 having SL-UA (mean age 62.0 y., 42.9% male, 19 SSL, 1 TSA, 1 SEC) and 35 having SL-IA (mean age 60.8 y., 62.9% male, 27 SSL, 1 TSA and 8 SEC). Both groups had similar time intervals between IBD diagnosis and SL diagnosis (p>0.05), and similar maximal therapeutic maintenance steps (p>0.05), as well as Mayo/SES-CD scores, serum C-reactive protein, hemoglobin, and albumin, and fecal calprotectin values at index and last colonoscopy (p>0.05).

**Conclusion(s):**

Patients with SL and IBD are not at higher risk of total metachronous advanced neoplasia than patients with SL and no IBD. SLs in IBD should be considered sporadic and undergo endoscopic resection and follow-up similar to non-IBD patients.

**Please acknowledge all funding agencies by checking the applicable boxes below:**

CIHR, Other

**Please indicate your source of funding;:**

Fonds de Recherche du Québec en Santé

**Disclosure of Interest:**

None Declared

